# GABPA is a master regulator of luminal identity and restrains aggressive diseases in bladder cancer

**DOI:** 10.1038/s41418-019-0466-7

**Published:** 2019-12-04

**Authors:** Yanxia Guo, Xiaotian Yuan, Kailin Li, Mingkai Dai, Lu Zhang, Yujiao Wu, Chao Sun, Yuan Chen, Guanghui Cheng, Cheng Liu, Klas Strååt, Feng Kong, Shengtian Zhao, Magnus Bjorkhölm, Dawei Xu

**Affiliations:** 1 Department of Urology, Shandong Provincial Hospital of Shandong University, Jinan, PR China; 2 Key Laboratory for Kidney Regeneration of Shandong Province, Jinan, PR China; 3 Department of Medicine, Division of Hematology, Bioclinicum and Center for Molecular Medicine, Karolinsk Institutet and Karolinska University Hospital Solna, Stockholm, Sweden; 4 School of Medicine, Shandong University, Jinan, PR China; 5 Central Research Laboratory, the Second Hospital of Shandong University, Jinan, PR China; 6 Department of Urology, The Third Hospital of Beijing University, Beijing, PR China; 7 Karolinska Institute-Shandong University Collaborative Laboratories for Cancer and Stem Cell Research, Jinan, PR China

**Keywords:** Metastasis, Preclinical research

## Abstract

TERT promoter mutations occur in the majority of glioblastoma, bladder cancer (BC), and other malignancies while the ETS family transcription factors GABPA and its partner GABPB1 activate the mutant TERT promoter and telomerase in these tumors. GABPA depletion or the disruption of the GABPA/GABPB1 complex by knocking down GABPB1 was shown to inhibit telomerase, thereby eliminating the tumorigenic potential of glioblastoma cells. GABPA/B1 is thus suggested as a cancer therapeutic target. However, it is unclear about its role in BC. Here we unexpectedly observed that GABPA ablation inhibited TERT expression, but robustly increased proliferation, stem, and invasive phenotypes and cisplatin resistance in BC cells, while its overexpression exhibited opposite effects, and inhibited in vivo metastasizing in a xenograft transplant model. Mechanistically, GABPA directly activates the transcription of FoxA1 and GATA3, key transcription factors driving luminal differentiation of urothelial cells. Consistently, TCGA/GEO dataset analyses show that GABPA expression is correlated positively with luminal while negatively with basal signatures. Luminal tumors express higher GABPA than do basal ones. Lower GABPA expression is associated with the *GABPA* gene methylation or deletion (especially in basal subtype of BC tumors), and predicted significantly shorter patient survival based on TCGA and our cohort of BC patient analyses. Taken together, GABPA dictates luminal identity of BC cells and inhibits aggressive diseases in BC by promoting cellular differentiation despite its stimulatory effect on telomerase/TERT activation. Given these biological functions and its frequent methylation and/or deletion, GABPA serves as a tumor suppressor rather than oncogenic factor in BC. The GABPA effect on oncogenesis is context-dependent and its targeting for telomerase inhibition in BC may promote disease metastasizing.

## Introduction

Bladder cancer (BC) is one of the most common urological malignancies worldwide [1]. The vast majority (>90%) of BC arise from the urothelium, so-called urothelial BC, which is composed of nonmuscle invasive (~60%) and muscle invasive (up to 30%) BCs (NMIBCs and MIBCs) [2–4]. NMIBCs undergo frequent recurrence, but up to 90% of patients survive more than 5 years, while most MIBC patients die from progressive or metastatic diseases within 5 years [2, 3]. To improve prognostication and eventually clinical managements, a number of groups have made efforts in the molecular taxonomy classification of BCs in the last few years [5–14]. Indeed, it has been shown that BCs, including both NMIBCs and MIBCs, can be largely categorized into luminal and basal subtypes based on their unique gene expression profiles, mimicking breast cancer. The luminal BC phenotype is in general characterized by the expression of differentiation transcription factors and markers of differentiation (FoxA1, GATA3, PPARγ, KRT20, etc.) [3, 13]. In contrast, the basal subtype is enriched with BC stem cell (BCSC) and mesenchymal-like biomarkers such as KRT14, KRT5, CD44, Twists, Snails, and among others [3]. Several lines of evidence have suggested that this molecular classification may contribute more to a precise prediction of BC outcomes and improvement of clinical interventions. However, the molecular mechanism(s) controlling luminal or basal phenotypes of BC remains poorly understood and barely explored.

The mammalian ETS family of transcription factors play critical roles in development, cell differentiation and oncogenesis, and consist of >30 members, including GA-binding protein A (GABPA) [15]. GABPA forms a heterotetramer complex [(GABPA/GABPB)_2_] with its partner GABPB1 or GABPB2 through which DNA binding and transcriptional regulation is achieved [16]. In addition, GABPA interacts with other transcription factors or co-activators (Sp1, PU.1, MITF, and CBP/P300) to regulate gene expression [16]. Through these direct and indirect activities, GABPA is broadly involved in physiological and pathological processes. For instance, GABPA is required for proliferation and differentiation of both myeloid and lymphoid cells, survival and self-renewal of hematopoietic stem cells (HSCs) [17–20]. Like many other ETS factors, GABPA also exhibits oncogenic activities and has been implicated in the pathogenesis of leukemia, prostate cancer, and other malignancies [15, 18, 19, 21–23]. More recently, GABPA and its partner GABPB1 were further identified as key transcription factors binding to the mutant *TERT* promoter [24, 25]. The TERT promoter mutation, widespread in many malignancies including BCs, glioblastomas, melanoma, thyroid carcinoma (TC), and others, creates de novo ETS-binding motifs through which the GABP complex promotes TERT transcription and subsequent telomerase activation in these mutation-carrying tumors [24, 25]. In BCs, this mutation is the most common genetic event and seen in up to 85% of primary tumors [26–32]. Li et al. found that the TERT promoter mutation preferably occurred in BCSCs (CD44 + KRT5 + KRT20−), and mutant TERT promoter-harboring BCSCs possessed much stronger ability to self-renew and to form tumors in nude mice [33]. Moreover, mutating the wild-type (wt) TERT promoter in normal bladder stem cells (SC, CD44 + KRT5 + KRT20−) is sufficient to drive their transformation [33]. Given the intimate relationship between GABP proteins and the mutant TERT promoter frequently present in BCs, we premise that GABPA may be required in the pathogenesis of basal BC subtype in which stem cell markers are enriched. However, we unexpectedly observed that GABPA facilitated luminal differentiation of BC by directly stimulating FoxA1 and GATA3 transcription, while its ablation leads to accelerated proliferation, stemness, drug resistance, and aggressiveness of BC cells. The present findings thus suggest that GABPA acts a tumor suppressor in BC.

## Materials and methods

### The Cancer Genome Atlas (TCGA) and GEO datasets

TCGA database were downloaded at cBioPortal in Oct. 2018. Additional datasets GSE32894, GSE48277, and GSE13705 were downloaded from the GEO website (http://www.ncbi.nlm.nih.gov/geo/). mRNA levels derived from these datasets are arbitrarily expressed as fragments per kilobase million (FPKM).

### Patients

One hundred and twelve patients with BC who underwent surgery at Shandong University Hospitals between 2006 and 2016 were included in the study. The tumor specimens were collected after surgery and paraffin embedded. In 12 of the patients, two slides were made from different parts of their tumors, and therefore, a total of 124 samples were analyzed for GABPA and FoxA1 expression using immunohistochemistry (IHC). The clinic-pathological data of BC patients are summarized in Table S1. Forty-five of these patients were followed up for 8 years and their clinical information is listed in Table S2. The study was approved by the Shandong University Second Hospital ethics committee and informed consent was obtained from all patients.

### Cell lines, cell culture, and TERT promoter sequencing

BC cell lines used in the present study included J82, SW1710, and HT1197, which were purchased from American Type Culture Collection (Manassas, VA). Cells were cultured in RPMI-1640 medium (Thermo Fisher Scientific, Waltham, MA) supplemented with 10% fetal bovine serum (FBS) (Thermo Fisher Scientific), 100 U/ml penicillin, 100 μg/ml streptomycin, and 4 mM l-glutamine. Cells were analyzed for mycoplasma infection every 6 months. All three cell lines harbor the C228T TERT promotor mutation, as determined by Sanger sequencing (Fig. S5). PCR and sequencing primers are listed in Supplementary Table S4.

### SiRNA transfection

GABPA siRNAs were from Thermo Fisher Scientific, and FoxA1 siRNAs from Integrated DNA technology (Coralville, Iowa). They were transfected into cells with Lipofectamine2000 (Thermo Fisher Scientific) according to the protocol provided by the manufacturer. Sequences for these siRNAs are listed in Supplementary Table S4.

### Cisplatin incubation and cell viability

Cells depleted of GABPA were incubated with cisplatin at different concentrations (6.25–25.0 µM) for 24 h followed by MTT treatment for additional 4 h at 37 °C. With dissolving the formazan, the spectrophotometric absorbance was determined using a microtiter enzyme-linked immunosorbent assay reader at 550 nm.

### BrdU incorporation assay

BrdU was added at the concentration of 50 μmol/L for 1 h. Cells were then fixed, permeabilized, and incubated with the BrdU antibody (1:1000, Sigma-Aldrich St. Louis, MO) followed by TRITC-conjugated secondary antibody (1:200, Santa Cruz), and finally stained with DAPI. A total of 100 cells were counted and photographed using Fluorescence microscopy (Nikon Eclipse 600) and percentages of positive cells were presented (mean ± SD).

### RNA extraction, reverse transcription, and quantitative PCR (qPCR)

Total RNA was extracted with Trizol-Reagent (Thermo Fisher Scientific), and reversely transcribed using the High Capacity cDNA Reverse Transcription Kit (Thermo Fisher Scientific). qPCR was performed in QuantStudio 7 Flex Real-Time PCR System using SYBR Green (Thermo Fisher Scientific). Levels of target mRNA (TERT, FoxA1, GATA3, GABPA, CDKN1A, and CDNK1B) were calculated based on the ΔCT values and normalized to human β2-M expression. Primers used in this study are documented in Supplementary Table S4.

### Western blot analysis

Cellular proteins were extracted using Pierce RIPA Buffer (Thermo Scientific) with 1% Phenylmethanesulfonyl fluoride (Sigma-Aldrich) and quantified with the DC Protein Assay (Bio-Rad). Thirty micrograms of proteins was separated in Mini-PROTEAN TGX Gels (Bio-Rad) and transferred to PVDF membranes using Trans-Blot Turbo Transfer Pack (Bio-Rad). Membranes were blocked with 5% nonfat milk diluted in TBST, and then incubated with primary antibodies and secondary antibodies before imaged with Clarity Max Western ECL Substrate (Bio-Rad, 1705062) and ChemiDoc MP Imaging System (Bio-Rad). Primary antibodies used for cell lines included anti-GABPA (1:1000 dilution, Santa Cruz, sc-22810), anti-FoxA1 (1:1000 dilution, Santa Cruz Biotechnology, Sc-514695), GATA3 (1:200 dilution, Abcam, ab14601), CDKN1A and 1B (1:1000 dilution, Santa Cruz Biotechnology), anti-OCT4 (1:500 dilution, Proteintech Group, Rosemont, IL, 11263-1-AP), anti-β-Actin (1:20,000 dilution, Santa Cruz Biotechnology, sc-47778), and anti-GAPDH (1:20,000 dilution, sc-47724 Santa Cruz Biotechnology). Secondary antibodies includes Goat Anti-Mouse IgG (H + L)-HRP Conjugate (Bio-Rad, 170-6516) and Goat Anti-Rabbit IgG (H + L)-HRP Conjugate (Bio-Rad, 170-6515).

### Flow cytometry

For cell cycle analyses, cells were fixed with 70% ethanol at +4 °C overnight and stained with RNAse A (0.5 μg)-containing Propidium Iodide (50 μg/ml). Cell cycle distribution was determined using flow cytometry with ModFit (BD Biosciences, Franklin Lakes, NJ). The assessment of BC stem cell marker CD44 was performed by staining cells with FITC-conjugated anti-CD44 Antibody (Biolegend, San Diego, #103022), and fluorescence signals were measured as the expression level of CD44. The FITC Annexin V Apoptosis Detection kit (BD Biosciences, San Jose, CA, #556547) was used to determine apoptosis.

### Clonogenic assay

Cells with different treatments (1000 cells/well) were seeded in six-well plates and incubated for 2 weeks. Cells were then fixed and stained with Giemsa. Colonies (>50 cells) were counted and photographed.

### Mono-spheroid formation assay

Cells (2000/well) were cultured in ultra-low attachment 96-well plates (Corning Life Sciences) with 100 μl RPMI-1640/10 mM HEPES serum-free medium supplemented with cocktails of following growth factors: 10 ng/mL bFGF (PeproTech Nordic, Stockholm, Sweden) and 20 ng/mL EGF (PeproTech Nordic). Fresh medium was supplemented every 3 days. Fifteen days later, the spheroid colonies were examined under light microscopy, counted, and photographed.

### Cellular invasion assays

Fifty microliters of matrigel (Corning Life Sciences, Flintshire, UK) was loaded to the bottom of the upper chamber, and cells (1.0 × 10^4^) were then seeded into the upper chamber. The low chamber contained RPMI-1640 medium with 20% FBS. Cells passing through the matrigel were stained with crystal violet, counted, and photographed 24 h later.

### Promoter activity assessment

The luciferase constructs harboring wt C228T and C250T *TERT* promoter sequences were kindly provided by Dr JF Costello (University of California, San Francisco) [24]. CDKN1A (pWWP from −2300 to +8) and CDKN1B (from −1358 to +32) reporter constructs harboring their promoter sequences were described [34]. FoxA1 and GATA3 promoter constructs were made by Shanghai Integrated Biotech Solutions Co. Ltd Putative GABPA-binding sites on the FoxA1 and GATA3 promoter regions were identified using the Consite software and its mutant variant made using a Site-Directed Mutagenesis Kits (Thermo Fisher Scientific). The above promoter reporters were transfected into J82 and SW1710 cells, or co-transfected with *GABPA* expression vectors. Cells were then harvested and luciferase activity was determined using a dual luciferase reporter assay system (Promega, Madison, USA). The target promoter-driven firefly luciferase activity was normalized to the renilla activity included in the kit.

### Chromatin immunoprecipitation (ChIP)

The SimpleChIP^®^ Enzymatic Chromatin IP Kit (Magnetic Beads, #9003, Cell Signaling Technology) was used according to the protocol provided. In brief, J82 and SW1710 cells were crosslinked with formaldehyde. Chromatin digestion was performed with Micrococcal Nuclease and analyzed by agarose gel. DNA concentration was assessed by Nanodrop (Thermo Fisher Scientific). The antibodies against GABPA (Abcam, Cambridge, UK, Cat# AB190747) and positive control (histone H3) antibodies were added into the digested samples and incubated overnight at 4 °C with rotation. Protein G Magnetic beads were used to precipitate the DNA-antigen–antibody complex followed by the elution of chromatin from Antibody/Protein G Magnetic Beads and reversal of cross-links. Spin columns were used to purify DNA and the collected DNA was amplified using PCR with specific primers (Supplementary Table S4).

### Immunohistochemistry (IHC)

Paraffin-embedded slides were deparaffinized and rehydrated followed by antigen retrieval using citric acid buffer. Endogenous peroxidase was deactivated by H_2_O_2_. Slides were blocked using 10% goat serum and incubated with the corresponding primary antibodies overnight at 4 °C. After incubation with secondary antibodies for 45 min at room temperature, DAB staining (Thermo Fisher Scientific) was used to detect the antigen–antibody binding. The primary antibodies used were: GABPA (Cell Signaling Technology) and FoxA1 (Santa Cruz Biotechnology). The slides were examined by two of the coauthors (YG and DX) and mean values of GABPA and FoxA1 positive cells were presented based on the results from two observers. For each slide, a total of 200 cells in two fields were analyzed.

### Nude mice and tumor cell injection

Six-week-old male athymic BALB/c nude mice were purchased from Beijing Vital River Laboratory Animal Technology Co., Ltd/Charles River Laboratories, Beijing, China, and used to evaluate the in vivo effect of GABPA on metastasis by randomly dividing them into two groups. J82 cells expressing ectopic GABPA (J82/GABPA) and control cells with empty vectors (J82/Control) were injected into nude mice via the tail vein. Mice were killed after 10 weeks and lungs/livers were collected for evaluation of tumor seeding or metastasis by using BOUNIS/hematoxylin and eosin (H&E) staining. The study was approved by Shandong University Second Hospital Ethics Committee.

### Statistical analyses

All statistical analyses were performed using IBM SPSS Statistics version 24 (IBM, Armonk, NY). Based on the distribution of data, Student’s *t* test, Mann–Whitney *U*-test, and Chi^2^-test or Fisher’s exact test were used for univariate analysis. Spearman’s Rank-Order Correlation was applied to determine correlation coefficient *ρ* and *P* value. OS and DFS were visualized with Kaplan–Meier plots. Survival analyses were performed with log-rank test. Levels of *GABPA* transcripts were classified into low and high groups using median separation. All the tests were two-sided and *P* values < 0.05 were considered as statistically significant.

## Results

### GABPA expression is highly enriched in luminal BC tumors

**Fig. 1:**
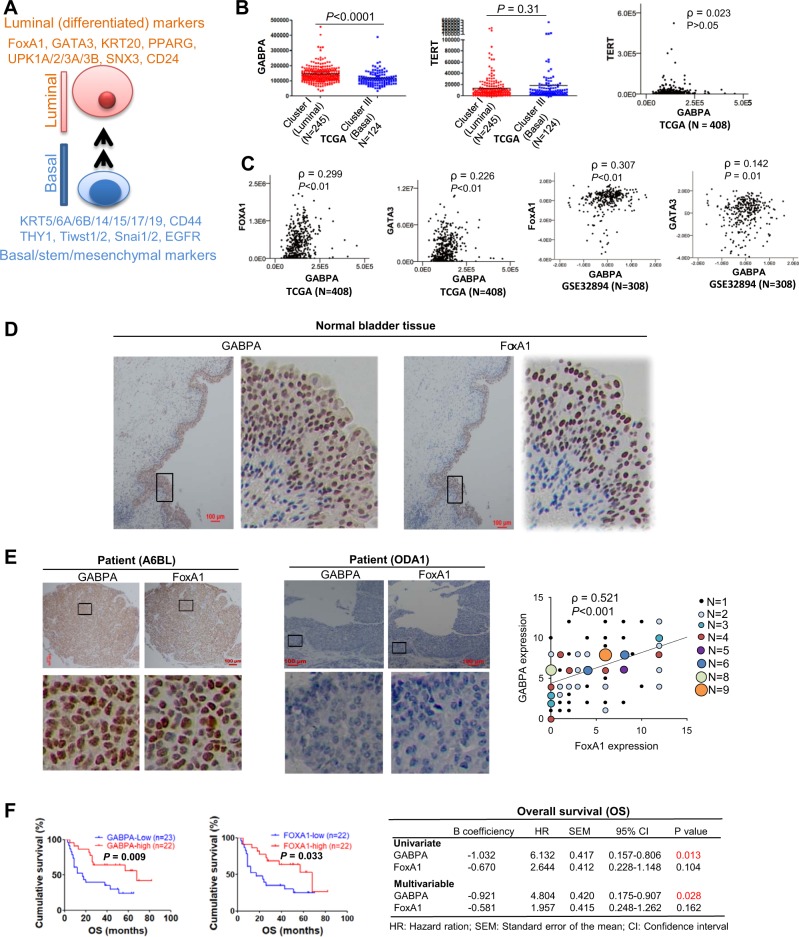
GABPA expression is higher in the luminal subtype and associated with luminal signature, and predicts patient survival in bladder cancer (BC). **a** Schematic diagram showing luminal and basal BCs and their gene expression signatures. **b** Differential GABPA expression between luminal and basal subtypes of BCs. The TCGA cluster I (luminal) and III (basal) were compared for their GABPA and TERT (Telomerase reverse transcriptase) mRNA expression. Their mRNA levels are expressed as transcript reads determined using RNA sequencing. TERT telomerase reverse transcriptase. **c** Positive correlation between GABPA and FoxA1/GATA3 mRNA expression in BCs. The TCGA and GSE32894 cohorts of BC patients were analyzed for the relationship of GABPA with FoxA1 and GATA3 transcript levels. **b**, **c** mRNA levels are arbitrarily expressed as FPKM (fragments per kilobase million). **d** Positive correlation between GABPA and FoxA1 protein expression in BC tumors. One hundred and twenty-four tumors from 112 patients with BC were analyzed for GABPA and FoxA1 protein levels using immunohistochemistry (IHC). Representative staining images (double positive and negative signals) were shown (Left and middle panels). Scale bars: 100 μm. The bottom panels: the enlarged areas of insets in the top panels. Right panel: the significant positive correlation between GABPA and FoxA1 expression as determined using IHC. **e** GABPA and FoxA1 expression as determined using IHC in adult human bladder. Shown are representatives of adjacent normal bladder tissues derived from patients with bladder cancer. GABPA signals are predominately localized in the bladder epithelium. Very similar expression profile of FoxA1 is shown here, too. **f** GABPA expression is associated with BC survival. Tumors derived from 45 BC patients were analyzed for their GABPA and FoxA1 expression using IHC and the medium level was used as a cutoff to separate low- and high-expression groups. GABPA and FoxA1 expression with survival association was determined by using univariate and multivariable analyses. OS overall survival

Luminal BCs express differentiation transcription factors and markers of differentiation whereas the basal tumor subtype is enriched with SC and mesenchymal-like biomarkers (Fig. 1a) [2, 3]. Given the role of GABPA in BCSCs together with its stimulatory effect on the mutant TERT promoter, we sought to determine whether more aggressive basal BC tumors express higher levels of GABPA mRNA. By analyzing the TCGA dataset, however, we unexpectedly found that GABPA mRNA was highly abundant in luminal tumors [Figs. 1b and S1A, luminal vs basal (mean ± SD): 143,858 ± 53,918 vs 115,668 ± 44,104, *P* < 0.0001]. TERT expression was analyzed simultaneously, and there was no significant difference between these two BC subtypes. The Spearman correlation test showed that GABPA and TERT expression was independent of each other (Fig. 1b).

### GABPA expression is correlated positively with luminal while negatively with basal BC expression signatures and predicts patient survival

We then sought to determine whether GABPA is associated with luminal or basal gene expression signatures (Fig. 1a). Because FoxA1 and GATA3 are two key transcription factors driving a luminal differentiation program [2, 35], their relation to GABPA was first examined. The positive correlation of GABPA expression with both FoxA1 and GTGA3 levels was observed in the TCGA and GES32894 cohorts [8, 9] (Fig. 1c). In addition, similar results were also obtained from two other available datasets (GES48277 and GES13507) [5, 7, 36] (Fig. S1b, c). We further analyzed other known biomarkers in the TCGA cohort, and a positive correlation between the expression levels of GABPA and luminal differentiation factors KRT20, PPARG, UPKs, SNX31, and CD24 was revealed (Fig. S2). In contrast, a significant negative correlation was found between GABPA and basal markers including SC and mesenchymal-like molecules (CD44, KRT5/6/14, EGFR, Snails, and Twists) (Fig. S2). Because Mo et al. [13] recently identified an 18 gene expression signature that accurately distinguishes between basal and luminal subtypes, we also analyzed the TCGA cohort to determine the relationship between these 18 genes and GABPA. In addition to the markers described above (FoxA1, GATA3, KRT5/14/20, UPK1A/1B/2/3A, EGFR, and CD44), this signature panel also included KRT8/15/17/18, THY1, STAT3, JAK2, and ITGA6. A strongly negative or positive correlation with GABPA expression was observed in 14 of 18 genes whereas only 4 of them (KRT8/15/18 and JAK2) were independent of GABPA (Figs. 1, S1 and S2, and data not shown).

GABPA protein expression was then assessed in primary tumors and adjacent normal bladder tissues derived from 112 BC patients using IHC. Adjacent nontumorous bladder tissues (from 64 patients) exhibited positive staining of both GABPA and FoxA1 predominately localized in the urothelium layer (Fig. 1d). We also performed GABPA staining on normal bladders derived from 2-month old mice (three subjects) and 16-day murine fetus (three subjects), respectively, and observed an identical distribution of GABPA-expressing cells (Fig. S3). In tumors from BC patients, GABPA and FoxA1 signals varied but their expression was highly correlated with each other (Fig. 1e, *ρ* = 0.512 and *P* < 0.001). There was no association between GABPA expression and patient age, gender, stage and grade, tumor size and number, and invasiveness in this cohort of patients (Table S1).

The relationship between GABPA expression and survival was further evaluated. Follow-up data were available in 45 of 112 BC patients in the present cohort and the median level of GABPA expression served as a cutoff to divide patients into high and low groups. The patient characteristics are listed in Table S2. The overall survival (OS) was significantly longer in patients with high-GABPA expression (Fig. 1f). Because FoxA1 is an established prognostic factor for BC, and its expression correlates closely with GABPA, we included FoxA1 analysis for comparison. As expected, lower FoxA1 expression correlated with shorter OS (Fig. 1f), however, the multivariable analysis showed that only GABPA, rather than FoxA1, predicted patient OS independently (Fig. 1f, right). To validate this finding, we did the same analysis of 408 BC patients in the TCGA cohort. OS was significantly longer in the high-GABPA patient group (Fig. S4), whereas there was no difference in disease-free survival (DFS) between two groups. Higher FoxA1 or GATA3 levels were similarly associated with longer OS but not DFS (Fig. S4). However, none of these factors independently predicts OS based on the multivariable test (Table S3).

### FoxA1 and GATA3, the key transcription factors responsible for luminal cell differentiation, are the direct downstream target genes of GABPA

**Fig. 2:**
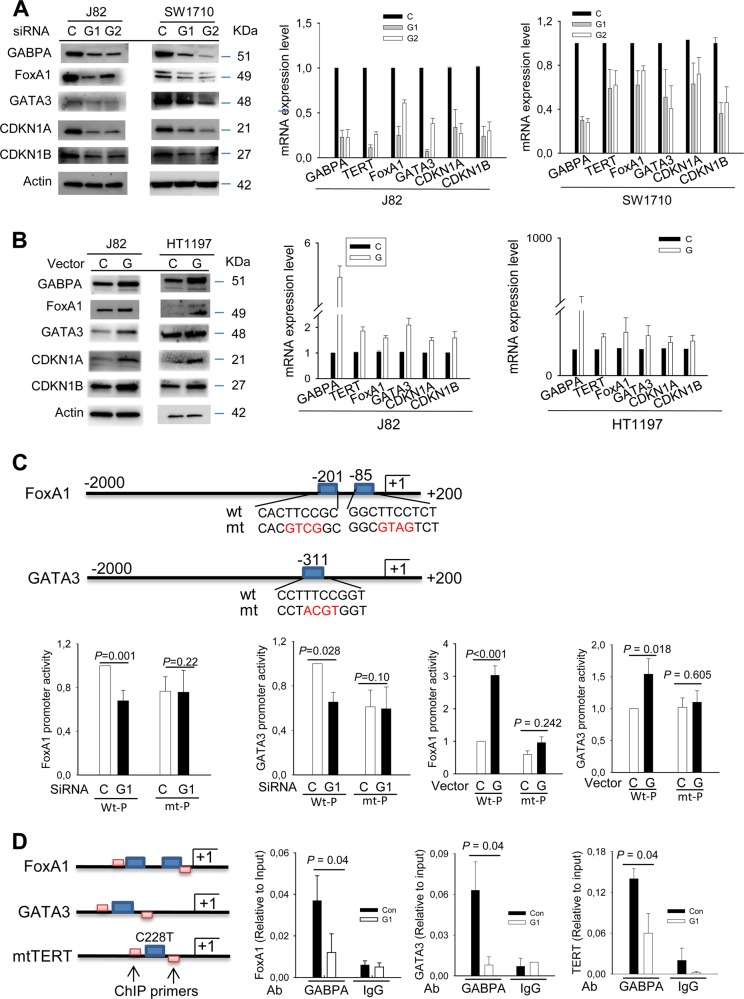
GABPA regulates FoxA1 and GATA3 expression at transcriptional levels. Three independent experiments were performed. Bars: SD. **a** Downregulation of FoxA1 and GATA3 expression and their targets in BC cells depleted of GABPA. J82 and SW1710 cells were treated with two different GABPA siRNAs for 48 h and then analyzed for target gene expression at mRNA and protein levels. **b** Enhanced expression of FoxA1 and GATA3 in BC cells with GABPA overexpression. Ectopic GABPA was introduced into J82 and HT1197 cells and the target gene expression was then determined. **c** GABPA regulation of FoxA1 and GATA3 promoter activity. The upper panel: schematic presentation of FoxA1 and GATA3 promoters and locations of putative GABPA-binding motifs. The mutation of putative GABPA-binding motifs is marked in red. The bottom panel: GABPA depletion leads to the decline in wt but not mt promoter activity, while its overexpression promotes wt but not mt promoter activity. **d** The GABPA occupancy on FoxA1 and GATA3 promoter regions. Chromatin immunoprecipitation (ChIP) was performed to determine the presence of GABPA on the FoxA1 and GATA3 promoters. The TERT promoter with C228T was used as a positive control. Left: schematics of the GABPA-binding sites and primer locations in the promoter regions of *TERT*, *FoxA1*, and *GATA3* genes. Right: qPCR assessment of GABPA bound-promoter sequences in J82 cells treated with control or GABPA siRNA. C Control siRNA or vector, G1 and G GABPA siRNA and expression vector, respectively

Because GABPA functions as a transcription factor whereas FoxA1 and GATA3 are the critical molecules driving luminal differentiation of both normal and malignant urothelial cells, we sought to probe whether GABPA directly regulates FoxA1 and GATA3 expression. Towards this end, we manipulated GABPA expression in BC-derived J82, SW1710 and HT1197 cells and then analyzed changes in FoxA1 and GATA3 mRNA and protein levels. J82 and SW1710 cells were transfected with GABPA-specific siRNAs to inhibit its expression. As shown in Fig. 2a, the abundance of FoxA1 and GATA3 mRNAs and proteins were greatly diminished in these GABPA-depleted J82 cells; and their decline similarly occurred in SW1710 cells, albeit to a lesser extent regarding mRNA levels. By contrast, the ectopic introduction of GABPA into J82 and HT1197 cells enhanced FoxA1 and GATA3 expression (Fig. 2b). Since all three cell lines harbor the C228T TERT promoter mutation, as revealed by Sanger sequencing analyses (Fig. S5A), we included TERT expression analyses. As expected, TERT expression was downregulated upon GABPA knockdown while upregulated in cells with ectopic GABPA expression (Fig. 2a, b). Moreover, luciferase activity driven by the mutant but not the wt TERT promoter was substantially inhibited by GABPA depletion while enhanced by its overexpression (Fig. S5B).

To further determine whether GABPA regulates the transcription of *FoxA1* and *GATA3* genes, we searched for canonical ETS/GABPA-binding motifs on their promoters, and a number of putative-binding sites were indeed identified (Fig. 2c). GABPA transfection stimulated FoxA1 and GATA3 promoter activity in SW1710 and HT1197 cells, while mutating the GABPA-binding sites led to a significant decline in their basal activity, and GABPA cotransfection was unable to further enhance the activity of the mutant promoters. These results suggest an essential role for GABPA in the transcription of *FoxA1* and *GATA3* genes. Finally, we performed ChIP assay to examine GABPA occupancy on these two promoter regions using J82 cells. The mutant (C228T) TERT promoter ChIP was included as a positive control. Figure 2d shows the GABPA signal enrichment on the FoxA1, GATA3, and TERT promoters, while its depletion led to its diminished presence in these regions.

### GABPA regulates proliferation of BC cells

**Fig. 3:**
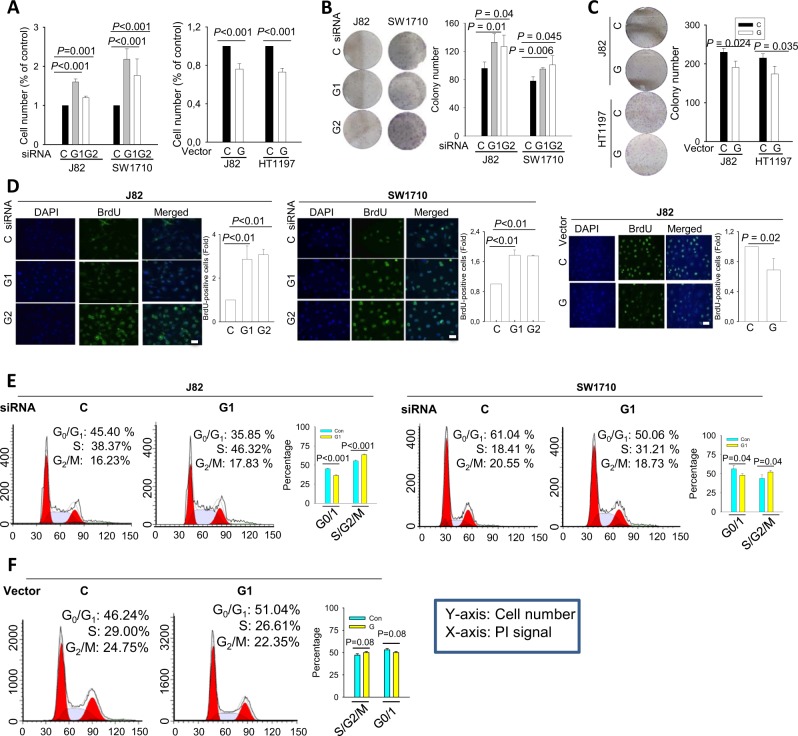
GABPA regulates BC cell proliferation. Three independent experiments were performed. Bars: SD. **a** Cell number changes resulting from the manipulation of GABPA expression. GABPA depletion accelerates while its overexpression slows down cell proliferation rates. **b** Enhanced clonogenic capabilities resulting from the GABPA depletion. J82 and SW1710 cells were treated with GABPA siRNAs for 48 h and colony formation then determined. **c** Inhibition of clonogenic capabilities mediated by GABPA overexpression. Control and GABPA-expressed J82 and HT1197 cells were compared for their ability to form colonies. **d** BrdU incorporation for the cellular replication assay. Shown are representative images and percentages of BrdU^+^ cells are calculated based on three independent experiments. Scale bars: 10 μm. **e** Cell cycle analyses of J82 and SW1710 cells with GABPA knockdown for 48 h. Shown are representative images and percentages of cells at G0/1 and S/G2/M are calculated based on three independent experiments. **f** Cell cycle analyses of J82 cells expressing ectopic GABPA. Shown are representative images and percentages of cells at G0/1 and S/G2/M are calculated based on three independent experiments. C Control siRNA, G1 and G2 GABPA siRNA1 and 2, G GABPA expression vector

It is well established that both FoxA1 and GATA3 induce CDKN1A and CDKN1B expression transcriptionally [37–39], and their decline in mRNA and protein levels were indeed coupled with downregulation of FoxA1 and GATA3 in GABPA-depleted cells (Fig. 2a, b). Because CDKN1A and CDKN1B are potent cyclin-dependent kinase inhibitors, we sought to investigate the potential effect of GABPA on cell proliferation. GABPA depletion led to accelerated proliferation of both J82 and SW1710 cells, as demonstrated by significantly increased cell numbers and clonogenic potential compared with those of the control siRNA-treated cells (Fig. 3a, b). Consistent with cell and colony-counting results, the BrdU incorporation experiments showed that more GABPA-depleted J82 (>2-fold) and SW1710 cells (>1.7-fold) were positive for BrdU signals (Fig. 3c, GABPA vs control siRNA in all: *P* < 0.01). Consistently, these cells in S and G2/M phases significantly increased coupled with reduced G0/1 upon GABPA knockdown, as determined using flow cytometry (Fig. 3e). In contrast, the ectopic expression of GABPA in J82 and HT1197 cells upregulated the abundance of CDKN1A and 1B (Fig. 2a), and moreover, the promoter activity of CDKN1A and 1B similarly increased in the same cells (Fig. S6). These cells proliferated more slowly and a 20% decrease in cell numbers was observed compared with their control counterparts within 48 h of incubation (Fig. 3b). In addition, these cells exhibited lower abilities to form colonies, reduced BrdU incorporation (Fig. 3c, d) and increased G0/G1 accumulation (Fig. 3f). We further determined the effect of GABPA on cellular survival, and slightly reduced apoptosis was observed in J82 cells overexpressing GABPA compared with their control counterparts (Fig. S7).

### GABPA controls invasion and stemness of BC cells

**Fig. 4:**
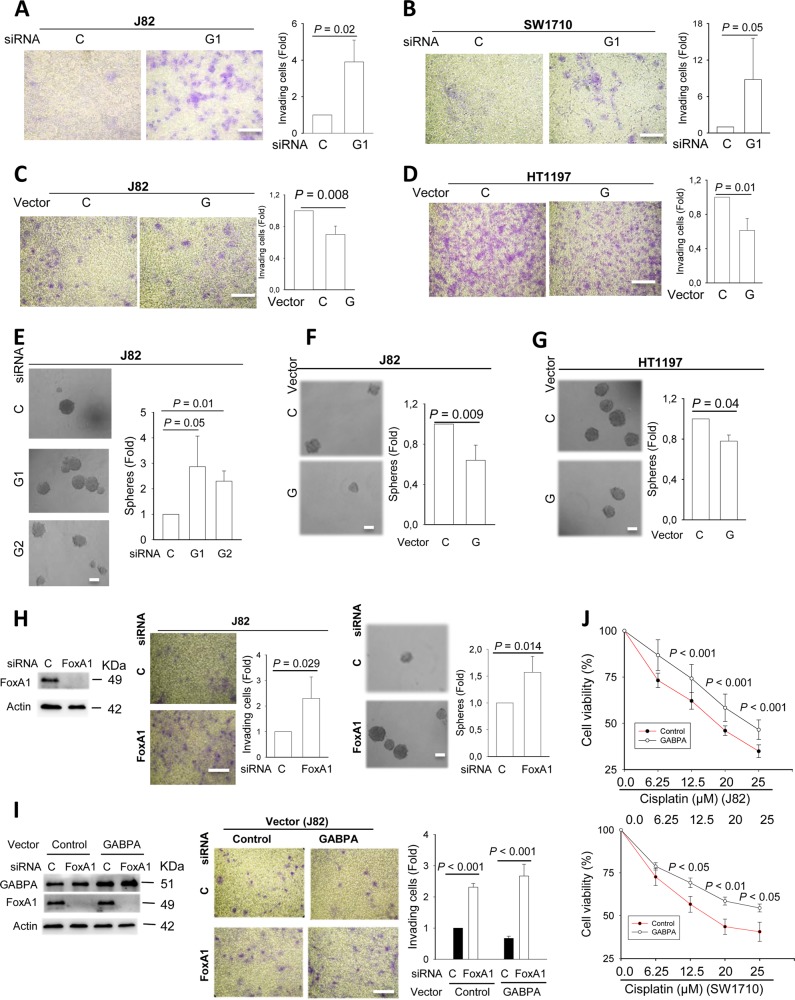
GABPA regulates invasion and stem cell phenotypes, and cisplatin sensitivity of BC cells. Three independent experiments were performed. Bars: SD. **a**, **b** GABPA depletion-mediated robust increases in BC cell invasion. J82 (left) and SW1710 cells (right) were treated with GABPA siRNA for 48 h and their invasive capability was evaluated using the matrigel assay. Cells passing matrigel were stained, counted, and photographed. **c**, **d** J82 and HT1197 cells were transfected with GABPA expression vectors and these cells were then analyzed as above. **e**, **f**, **g** GABPA regulation of BC stem cell self-renewal. Left panel: GABPA was knocked down in J82 cells using its specific siRNAs and sphere formation was then assessed. Middle and right panels: J82 and HT1197 cells with ectopic GABPA expression were assessed for their sphere formation. **h** FoxA1 effect on invasive and stem cell phenotypes of BC cells. J82 cells were depleted of FoxA1 expression using its specific siRNA and assessed for their abilities of invasion and self-renewal. **i** The abolishment of GABPA-mediated inhibition of J82 cell invasion via FoxA1 depletion. Control and GABPA overexpressed J82 cells were treated with FoxA1 siRNA and invasion then evaluated. **j** GABPA depletion-mediated cisplatin resistance to BC cells. J82 and SW1710 cells were treated with two different GABPA siRNAs and then incubated with various concentrations of cisplatin. Cell viability was determined using the MTT assay. Scale bars: 20 μm. C: Control; G1 and G2, GABPA siRNA1 and 2; G: GABPA expression vector

Having defined the promoting effect of GABPA on BC luminal differentiation, we hypothesize that its inhibition is associated with the acquisition of SC and aggressive phenotypes in BC cells. J82 cells were transfected with GABPA siRNA or expression vectors to manipulate its expression, and their invasion and self-renewal capabilities were then compared. GABPA depletion led to robustly increased J82 cells passing through matrigel, and these cells generated more and bigger spheres (Fig. 4a, e). By contrast, a significant decline in invaded cells and sphere numbers were observed in J82 cells ectopically expressing GABPA (Fig. 4c, f). The invasive potential was also elevated in SW1710 cells treated with GABPA siRNA (Fig. 4b), while the GABPA effect on self-renewal could not be evaluated due to their inability to generate spheres. We further analyzed HT1197 cells with GABPA overexpression, and a decrease in invasion and sphere formation was similarly observed (Fig. 4d, g).

As GABPA directly regulates FoxA1 expression, we sought to determine whether FoxA1 knockdown mimics the scenario observed in GABPA-depleted cells above. As shown in Fig. 4h, invasion and self-renewal ability was significantly augmented in J82 cells treated with FoxA1 siRNA. To directly probe whether FoxA1 is involved in the GABPA-regulated BC invasiveness, we knocked down foxA1 in J82 cells expressing ectopic GABPA, and then assessed their invasion. FoxA1 depletion not only totally erased the inhibitory effect of GABPA, but also led to a robust increase in invasive cells (Fig. 4i).

We further evaluated whether the observed effect of GABPA above resulted from its regulation of stem cell factors. OCT4 expression was thus determined in BC cells with GABPA manipulation. As shown in Fig. S8, Upregulated OCT4 levels were observed in GABPA-depleted J82 and SW1170 cells while its diminished expression occurred in J82 and HT1197 cells expressing ectopic GABPA. Another key BC stem cell marker CD44 was also assessed in J82 cells, and GABPA overexpression inhibited whereas its knockdown enhanced CD44 expression significantly (Fig. S8).

### GABPA inhibition confers resistant to cisplatin in BC cells

Cisplatin-based chemotherapy is the standard adjuvant treatment for BC, but resistance development remains a major challenge and the underlying mechanism is unclear. Given all the observations above, we thus probed whether GABPA expression was involved in cisplatin resistance. For this purpose, J82 and SW1710 cells depleted of GABPA were incubated with cisplatin at various concentrations. The MTT assessment revealed that the viability of cells with GABPA knockdown in the presence of cisplatin was significantly higher compared with their control counterparts (Fig. 4j).

### GABPA overexpression inhibits in vivo metastasis of BC cells in a xenograft mouse model

**Fig. 5:**
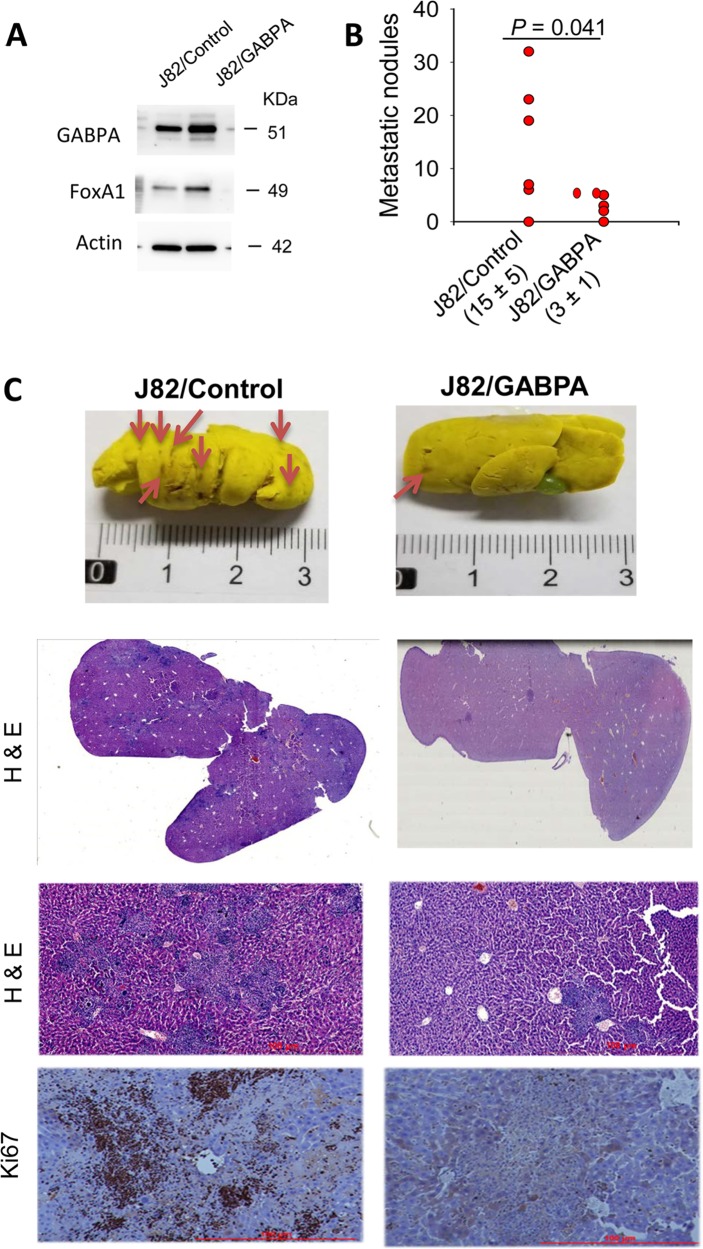
GABPA overexpression inhibits in vivo colonization/metastasis of J82 cells in a mouse model. **a** J82 subline stably expressing ectopic GABPA (J82/GABPA) were made and high-GABPA expression demonstrated using immunoblotting. **b** Control (J82/Control) and J82/GABPA cells were injected into nude mice (6/group) via the tail vein, respectively, and their livers were examined for tumor colony numbers 10 weeks later. Tumor numbers: Mean ± SD. **c** The representative livers (top panel). Mouse livers were stained by using BOUNIS solution, and tumor colonies exhibited dark color (indicated by arrows) while the rest part of livers were yellow. Lower panels: H&E and immunohistochemical staining (Ki67) of tumor nodules from the livers above. Scale bars: 100 μm

We further sought to determine whether GABPA expression affects in vivo metastasis of BC cells in a mouse model. J82 cells with ectopic GABPA expression (J82/GABPA) and their control counterparts (J82/Control) were injected into two groups of nude mice (6/group) via the tail vein, respectively. Mice were killed 10 weeks later, and their lungs and livers were examined for tumor cell seeding. Both J82/Control and J82/GABPA cells formed metastatic nodules in mouse livers but rarely in lungs; However, the number of tumor colonies were substantially fewer in livers from mice harboring J82/GABPA cells (J82/Control vs J82/GABPA: 15 ± 5/liver vs 3 ± 1/liver, *P* *=* 0.041) (Fig. 5a, b). IHC analyses showed that J82/Control tumors were more strongly positive for Ki67, in accordance with in vitro observations (Fig. 5c).

### The GABPA gene methylation and deletion, occurring in BC tumors, is associated with its downregulation or silence

**Fig. 6:**
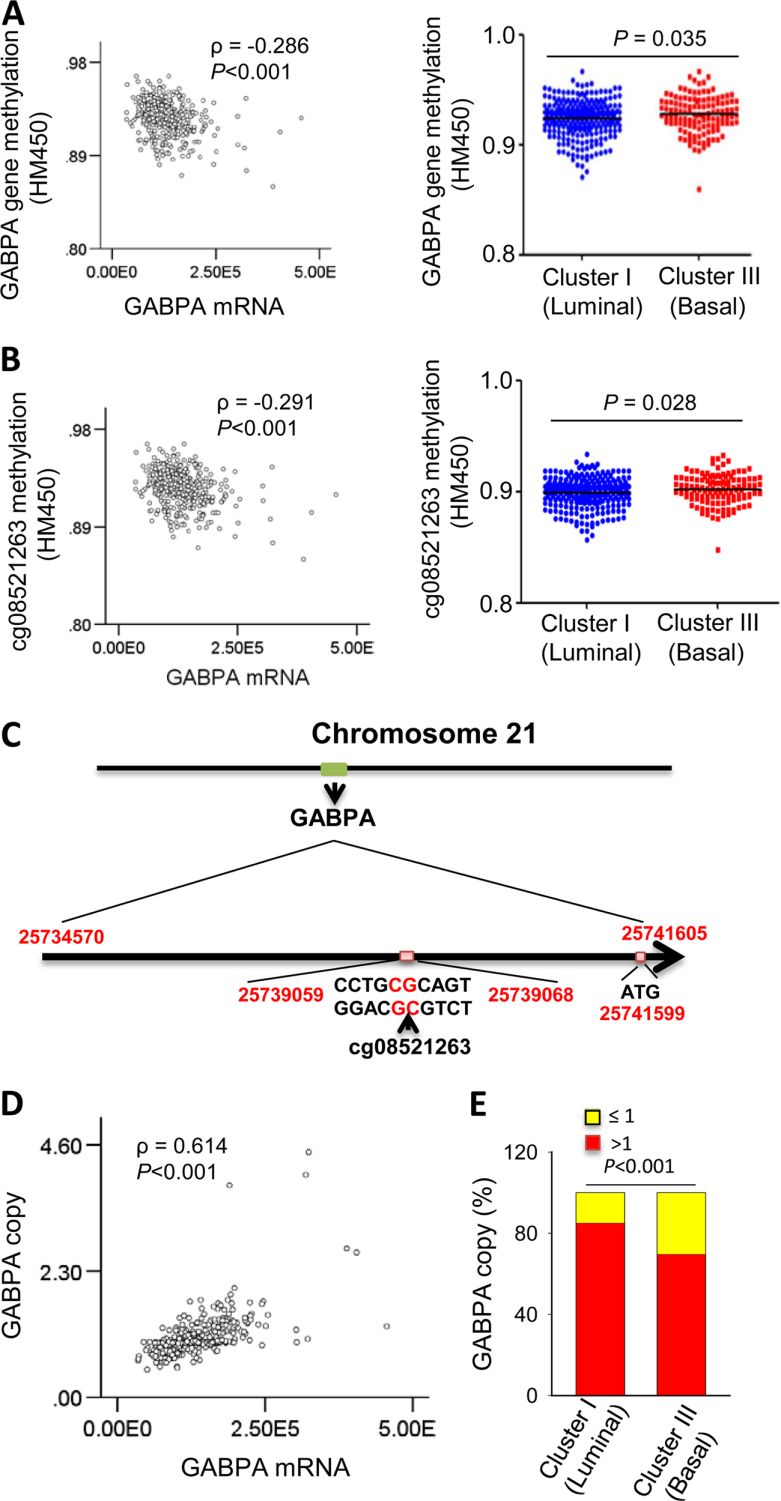
GABPA downregulation is associated with its gene methylation and/or deletion in bladder cancer (BC). The analysis was performed on the TCGA cohort of 412 patients with BC. **a** Left: the negative correlation between GABPA mRNA expression and gene methylation. Right: higher GABPA methylation in basal subtype of BC, as determined using Illumina HumanMethylation450 BeadChip. **b** Left: the negative correlation between GABPA mRNA expression and methylated CpG of cg08521263 at the *GABPA* allele (as detected by Illumina HumanMethylation450 BeadChip). Right: higher methylated CpG at cg08521263 in basal subtype of BC. **c** The schematic expression of the cg08521263 location at the *GABPA* allele. **d** The reduced GABPA expression resulting from its gene copy loss. The *GABPA* gene copy number and mRNA expression was positively correlated in the TCGA cohort of BC patients (Left). **e** More frequent loss of the *GABPA* gene in the basal subtype of BC

Finally, we sought to probe how GABPA is regulated in BCs. It is evident from all these observations that GABPA exhibits a tumor suppressive function in BC. Because tumor suppressors are generally silent by DNA methylation and/or genetic deletion/mutation, we determined whether this was the case for the downregulated GABPA expression in BCs. For this purpose, the TCGA dataset containing a cohort of 412 BC tumors was analyzed and the obtained results demonstrate that: (i) GABPA mRNA expression was negatively correlated with its allele methylation level (*P* < 0.001) (Fig. 6a). Moreover, we identified one single methylated CpG at cg08521263 of the *GABPA* allele played a predominant role in its silence (Fig. 6b, c); And (ii) 30% of BC tumors had a *GABPA* gene deletion and tumors with *GABPA* loss expressed significantly lower GABPA transcripts (Fig. 6d). Moreover, both methylation and deletion occurred more frequently in basal type of BCs (Fig. 6a, b, e). In addition, *GABPA* gene mutations occurred in 6 of 412 TCGA cohort patients (Fig. S9).

## Discussion

**Fig. 7:**
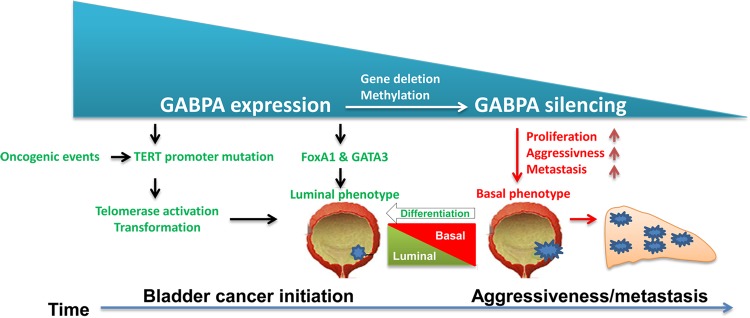
The context-dependent oncogenic and tumor suppressive role of GABPA in bladder cancer (BC). GABPA *trans*-activates mutant TERT promoter and thereby activates telomerase, which is required for the initiation of BC pathogenesis. In the meanwhile, GABPA inhibits BC aggressiveness and metastasis by promoting luminal differentiation. Abnormal *GABPA* gene methylation or deletion might occur, which leads to downregulated GABPA expression, thereby promoting BC progression

Our unprecedented discovery presented herein reveals a novel role of GABPA in regulating luminal differentiation of BC cells. In this manner, GABPA restrains proliferation, stemness, invasion, and drug resistance of BC cells. Thus, our results define an important impact of GABPA on the pathogenesis and progression of BCs (Fig. 7). Because FoxA1 and GATA3 transcription factors play a central role in the differentiation of urothelial cells, and their high expression is a key feature of the luminal subtype of BCs [5–14], we sought to elucidate whether and how GABPA dictates luminal identity or differentiation of BC cells by analyzing the relationship between GABPA and FoxA1/GATA3. It is evident from our results that both FoxA1 and GATA3 are the direct downstream targets of GABPA. Consistent with its association with the luminal phenotype of BCs, GABPA expression predicted significantly longer survival of BC patients.

A strong negative effect of GABPA on BC cell proliferation is unexpected. Yang et al. previously reported that GABPA was absolutely required for cell cycle entry of murine fibroblasts [40]. Mechanistically, GABPA induces the expression of its target gene *SKP2*, thereby leading to degradation of CDKN1A and B [40]. In addition, GABPA or GABPB1 was also shown to facilitate proliferation and/or survival of murine embryonal stem cells, hematopoietic stem cells, and T and B lymphocytes [17–20]. These observations are in sharp contrast to our observations. In BC cells, GABPA inhibition and overexpression led to proliferation acceleration and slowing down, respectively. Moreover, our results unequivocally confirm that GABPA regulates CDKN1A and B expression at a transcriptional level. GABPA thus exerts opposite effects on cell cycle progression via different mechanisms between human and murine cells, suggesting species-dependent effects. In addition, our data show that GABPA has a negligible impact on apoptosis of BC cells.

The present study reveals a dual role of GABPA in BC pathogenesis. On the one hand, given the widespread presence of TERT promoter mutations (up to 85%) in BCs and a stimulatory effect of GABPA on the mutant promoter, GABPA should be essential to TERT induction and telomerase activation in the malignant transformation of urothelial cells. TERT/telomerase contributes to multicancer hallmarks, and it not only confers malignant cells an immortal phenotype via telomere stabilization, but also promotes proliferation/survival, stemness, invasion, and drug resistance of cancer cells via telomere lengthening-independent mechanisms, thereby driving tumor progression including metastatic growth [26, 41–45]. On the other hand, GABPA activates the transcription of its downstream targets FoxA1 and GATA3 to promote luminal differentiation of BC cells, thereby restraining the development of undifferentiated, aggressive BCs. Despite significantly diminished TERT expression in mutant TERT promoter-carrying BC cells upon GABPA knockdown, these cells exhibited augmented proliferation, stemness, and invasive capacities, indicating that GABPA itself has a predominant role in suppressing BC aggressive state independently of TERT expression. This same scenario was recently observed in TC cells, too [46]. However, Mancini et al. [25] showed that mutant TERT promoter-harboring glioblastoma cells exhibited decreased TERT expression coupled with impaired proliferation upon GABPB1L depletion within 48 h and longer incubation time triggered telomere shortening/dysfunction and attenuated tumorigenic ability of these cells. Of note, a high frequency of TERT promoter mutations occurs in BC, melanoma, and TC, as seen in glioblastoma [25, 27, 47]. Therefore, the relationship between the mutant TERT promoter and GABPA is more complicated than expected, and GABPA may act as either a tumor-driver or suppressor in context-dependent manners.

Aberrant DNA methylation and genetic deletions are key mechanisms for the silencing of tumor suppressor genes in cancer. Interestingly, methylation and loss at the *GABPA* locus occurred widely in BC tumors, which are highly correlated with its downregulation. More specifically, we identified the methylation of a single CpG at cg08521263 as a key event affecting GABPA expression. In addition, the coding region mutation of the *GABPA* gene occurs in 1% of tumors, and based on our preliminary analyses, these mutations may have loss-of-function consequences. Conceivably, the genetic methylation/deletion/mutation-mediated GABPA repression promotes an undifferentiated state, thereby leading to the development of stem cell trait, aggressive phenotype and therapy resistance of BC cells, and eventual disease progression. Therefore, the assessment of GABPA expression and epigenetic/genetic alterations may have clinical implications. Since the GABPA methylation and copy loss is more frequently observed in the basal subtype of BCs, it is worthy of evaluating whether these alterations predict BC progression. The recent comprehensive analyses uncovered the clustering of genomic aberrations, and mutation and expression profiles in different subgroups of NIMBCs, and the loss of the TSC1 tumor suppressor is implicated in the disease progression [48].

In summary, GABPA functions as a master regulator of BC luminal identity by directly stimulating FoxA1 and GATA3 transcription. Because FoxA1 and GATA3 are required for the differentiation of urothelial cells, BC cells depleted of GABPA display accelerated proliferation, increased stemness or more immature status, and invasive properties, and lower sensitivity to cisplatin. Therefore, GABPA may serve as a tumor suppressor in BCs and a useful biomarker in BC prognostication and management decisions. Finally, the GABPA function may be species- and context-dependent, and it is thus essential to thoroughly delineate the full spectrum of roles for GABP factors in given cancer types for precision oncology.

## Supplementary information

Table S1

Table S2

Table S3

Table S4

Fig. S1

Fig. S2

Fig. S3

Fig. S4

Fig. S5

Fig. S6

Fig. S7

Fig. S8

Fig. S9
