# Supplementary material for: GABPA is a master regulator of luminal identity and restrains aggressive diseases in bladder cancer
Source: Cell Death Differ. 2019 Dec 4;27(6):1862–77. doi: 10.1038/s41418-019-0466-7 (PMC7244562; doi:10.1038/s41418-019-0466-7)
Supplement: Supplementary file 1 — Table S1 [file 41418_2019_466_MOESM1_ESM.doc]

| **Table S1. Clinicopathologic characteristics of 112 patients with bladder cancer** | | | | | |  |
| --- | --- | --- | --- | --- | --- | --- |
| **and relation to GABPA/FoxA1 expression** | | | | |  |  |
| **Variable** | N | GABPA (IHC) | *P* value | FoxA1 (IHC) | *P* value |  |
| **Sex** |  |  |  |  |  |  |
| Male | 91 | 6.07 ± 2.89 | 0.913 | 4.40 ± 3.83 | 0.284 |  |
| Female | 21 | 6.14 ± 2.95 |  | 5.38 ± 3.56 |  |  |
| **Age at diagnosis** |  |  |  |  |  |  |
| Mean ± SD | 65.29 ± 9.61 |  |  |  |  |  |
| Median (range) | 67.5（42 - 85） |  |  |  |  |  |
| **TNM stage** |  |  |  |  |  |  |
| <T2 | 9 | 7.22 ± 3.38 | 0.396 | 6.22 ± 3.53 | 0.118 |  |
| ≥T2 | 69 | 6.46 ± 2.38 |  | 4.25 ± 3.52 |  |  |
| Low | 12 | 6.75 ± 4.16 | 0.709 | 6.5 ± 4.58 | 0.2 |  |
| High | 64 | 6.42 ± 2.47 |  | 4.91 ± 3.79 |  |  |
| **Invasion** |  |  |  |  |  |  |
| NMIBC | 11 | 6.82 ± 3.76 | 0.437 | 7.55 ± 4.68 | 0.015 |  |
| MIBC | 64 | 6.08 ± 2.74 |  | 4.47 ± 3.61 |  |  |
| **Tumor size (cm)** |  |  |  |  |  |  |
| < 3 | 21 | 5.24 ± 3.28 | 0.165 | 4.14 ± 3.94 | 0.63 |  |
| ≥ 3 | 88 | 6.22 ± 2.78 |  | 4.59 ± 3.79 |  |  |
| **Tumor number** |  |  |  |  |  |  |
| Single | 93 | 6.17 ± 2.53 | 0.264 | 4.39 ± 3.63 | 0.742 |  |
| Multiple | 15 | 5.27 ± 4.64 |  | 4.73 ± 4.57 |  |  |
