# Supplementary material for: GABPA is a master regulator of luminal identity and restrains aggressive diseases in bladder cancer
Source: Cell Death Differ. 2019 Dec 4;27(6):1862–77. doi: 10.1038/s41418-019-0466-7 (PMC7244562; doi:10.1038/s41418-019-0466-7)
Supplement: Supplementary file 2 — Table S2 [file 41418_2019_466_MOESM2_ESM.doc]

| **Table S2. Clinic-pathologic characteristics of 45 BC patients** | | |
| --- | --- | --- |
| **Variable** | **N** |  |
| **Sex** |  |  |
| Male | 37 |  |
| Female | 8 |  |
| **Age at diagnosis** |  |  |
| Mean ± SD | 70.0 ± 9.0 |  |
|  |  |  |
| **TNM stage** |  |  |
| <T2 | 8 |  |
| ≥T2 | 34 |  |
| **Grade** |  |  |
| Low | 2 |  |
| High | 43 |  |
| **Invasion** |  |  |
| NMIBC | 3 |  |
| MIBC | 40 |  |
| **Tumor size (cm)** |  |  |
| < 3 | 8 |  |
| ≥ 3 | 35 |  |
| **Tumor number** |  |  |
| Single | 39 |  |
| Multiple | 4 |  |
|  |  |  |

BC: Bladder cancer
