# Supplementary material for: GABPA is a master regulator of luminal identity and restrains aggressive diseases in bladder cancer
Source: Cell Death Differ. 2019 Dec 4;27(6):1862–77. doi: 10.1038/s41418-019-0466-7 (PMC7244562; doi:10.1038/s41418-019-0466-7)
Supplement: Supplementary file 3 — Table S3 [file 41418_2019_466_MOESM3_ESM.doc]

| **Table S3. Multivariateanalyses of the association between survival and GABPA, FoxA1 and GATA3 expression in the TCGA cohort** | | | | | | | | | | |  |
| --- | --- | --- | --- | --- | --- | --- | --- | --- | --- | --- | --- |
|  | **Overall survival** | | | | |  | **Disease-free survival** | | | | |
|  | B coefficient | HR | Std.Err. | 95% CI | P value |  | B coefficient | HR | Std.Err. | 95% CI | P value |
| **Univariate analyses** |  |  |  |  |  |  |  |  |  |  |  |
| GABPA mRNA | -0,326 | 4,673 | 0,151 | 0.537-0.970 | 0.004 |  | -0,241 | 2,041 | 0,169 | 0.564-1.094 | 0.153 |
| FOXA1 mRNA | -0,464 | 9,219 | 0,153 | 0.466-0.848 | 0.002 |  | -0,130 | 0,593 | 0,169 | 0.630-1.223 | 0.441 |
| GATA3 mRNA | -0,354 | 5,458 | 0,152 | 0.521-0.945 | 0.019 |  | -0,245 | 2,088 | 0,169 | 0.562-1.091 | 0.148 |
|  |  |  |  |  |  |  |  |  |  |  |  |
| **Multivariate analyses** |  |  |  |  |  |  |  |  |  |  |  |
| GABPA mRNA | -0,237 | 2,360 | 0,154 | 0.583-1.068 | 0.124 |  | -0,208 | 1,462 | 0,172 | 0.579-1.138 | 0.227 |
| FOXA1 mRNA | -0,347 | 3,599 | 0,183 | 0.493-1.012 | 0.058 |  | 0,034 | 0,028 | 0,205 | 0.692-1.547 | 0.867 |
| GATA3 mRNA | -0,129 | 0,507 | 0,181 | 0.217-1.253 | 0.476 |  | -0,228 | 1,233 | 0,205 | 0.532-1.191 | 0.267 |
|  |  |  |  |  |  |  |  |  |  |  |  |
