# Supplementary material for: GABPA is a master regulator of luminal identity and restrains aggressive diseases in bladder cancer
Source: Cell Death Differ. 2019 Dec 4;27(6):1862–77. doi: 10.1038/s41418-019-0466-7 (PMC7244562; doi:10.1038/s41418-019-0466-7)
Supplement: Supplementary file 4 — Table S4 [file 41418_2019_466_MOESM4_ESM.doc]

| **Table S4. Sequences of primers, siRNAs and plasmids used in the study.** | |  |
| --- | --- | --- |
| **Target** | **Direction** | **Sequence** |
| **Sanger sequencing** |  |  |
| *TERT* promoter mutation | Forward | 5'-CACCCGTCCTGCCCCTTCACCTT-3' |
|  | Reverse | 5'-GGCTTCCCACGTGCGCAGCAGGA-3' |
|  |  |  |
| **Quantitative Real-time PCR (SYBR Green)** |  |  |
| *TERT* | Forward | 5’-CGGAAGAGTGTCTGGAGCAA-3’ |
|  | Reverse | 5’-GGATGAAGCGGAGTCTGGA-3’ |
| *GABPA* | Forward | 5’- AAGAACGCCTTGGGATACCCT-3’ |
|  | Reverse | 5’- GTGAGGTCTATATCGGTCATGCT-3’ |
| *FoxA1* | Forward | 5’-ggt ggc tcc agg atg tta gga-3’ |
|  | Reverse | 5’-ggt cat gta ggt gtt cat gga gt-3’ |
| *GATA3* | Forward | 5’-cgg agg agg tgg atg tgc ttt-3’ |
|  | Reverse | 5’-gcc agg gta ggg atc cat ga-3’ |
| *CDKN1A* | Forward | 5'-GCGACTGTGATGCGCTAAT-3' |
|  | Reverse | 5'-TAGGGCTTCCTCTTGGAGAA-3' |
| *CDKN1B* | Forward | 5’-ATGTCAAACGTGCGAGTGTCTAA-3’ |
|  | Reverse | 5’-TTACGTTTGACGTCTTCTGAGG-3’ |
| *β2-M* | Forward | 5’-CAGGTCAGTTGCAGTTTCAGCA-3’ |
|  | Reverse | 5’-CATCTTCAAACCTCCATGATG-3’ |
| **ChIP primers** |  |  |
| *FoxA1* | Forward | 5’-GGC ACT CAA GCG ACG TAA GA-3’ |
|  | Reverse | 5’-CCC AAC GCC ACC CGG GCG A-3’ |
| *GATA3* | Forward | 5'-GTCCACCCGACCCGAATGAA-3' |
|  | Reverse | 5'-CCGGCACAAGGAAACTGCA-3' |
| *TERT* | Forward | 5'-CACCCGTCCTGCCCCTTCACCTT-3' |
|  | Reverse | 5'-GGCTTCCCACGTGCGCAGCAGGA-3' |
|  |  |  |
| **siRNA** |  |  |
| GABPA (G1) |  | 5'-GGAGCUGAUAGAAAUUGAGAUUGAU-3' |
| GABPA (G2) |  | 5'-GCAGAGUGCACAGAAGAAAGCAUUG-3' |
| FoxA1 |  |  |
|  |  |  |
| **Constructs** |  |  |
| pGL3-FoxA1-Promoter reporter with and without GABPA site mutation | Shanghai Integrated Biotech Solutions Co.,Ltd | |
| pGL3-GATA3-Promoter reporter with and without GABPA site mutation | Shanghai Integrated Biotech Solutions Co.,Ltd | |
| GABPA expression plasmid | Shanghai Integrated Biotech Solutions Co.,Ltd | |
| CDKN1 and CDKN1B-promoter reporters | Provided by Drs. SH Juan and Y Sowa | |
| TERT promoter reporters (wt, C228T and C250T mutation) | provided by Dr. JF Costello |  |
