# Supplementary figures and images for: GABPA is a master regulator of luminal identity and restrains aggressive diseases in bladder cancer
Source: Cell Death Differ. 2019 Dec 4;27(6):1862–77. doi: 10.1038/s41418-019-0466-7 (PMC7244562; doi:10.1038/s41418-019-0466-7)

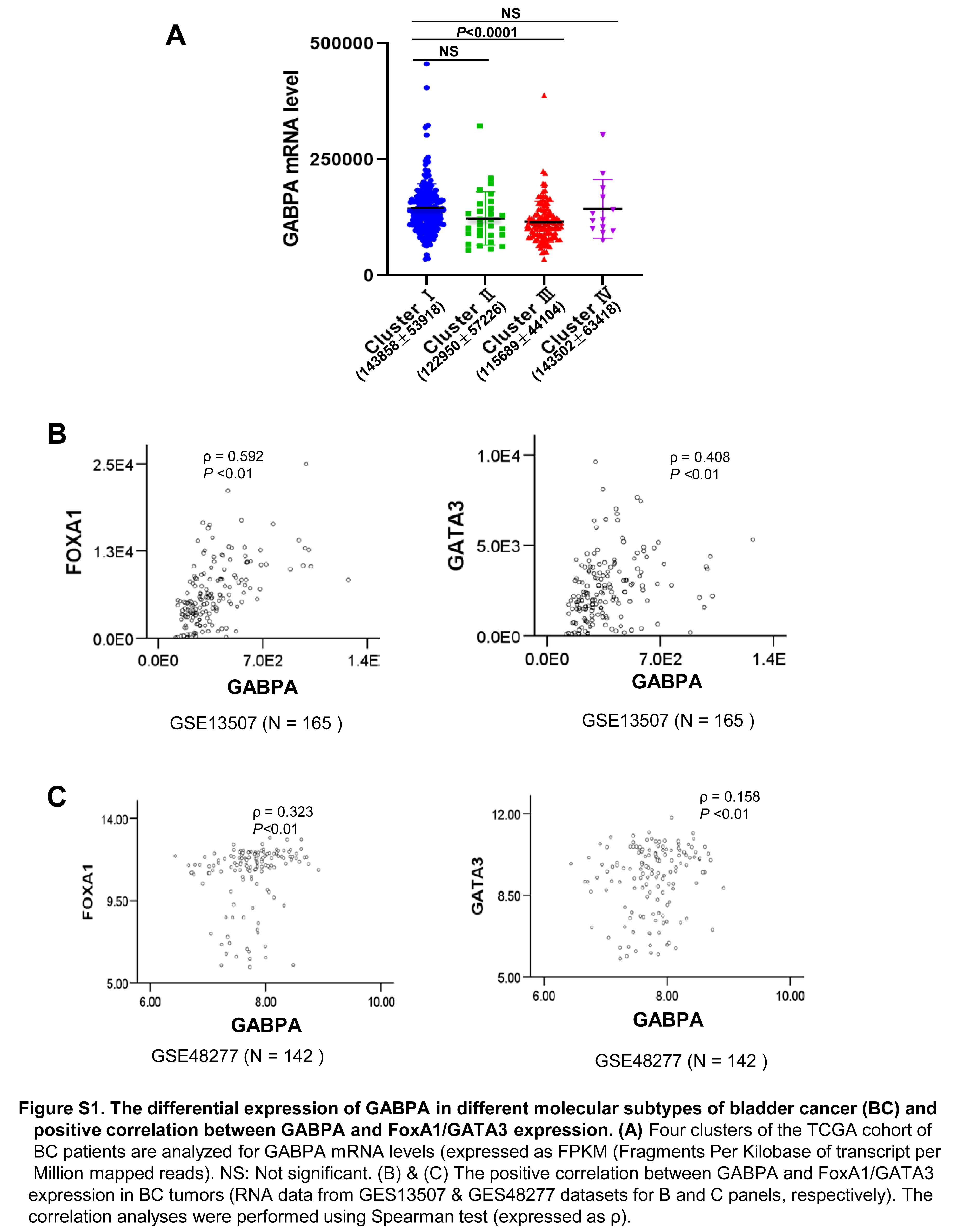

Supplement: Supplementary file 5 — Fig. S1 [file 41418_2019_466_MOESM5_ESM.png]

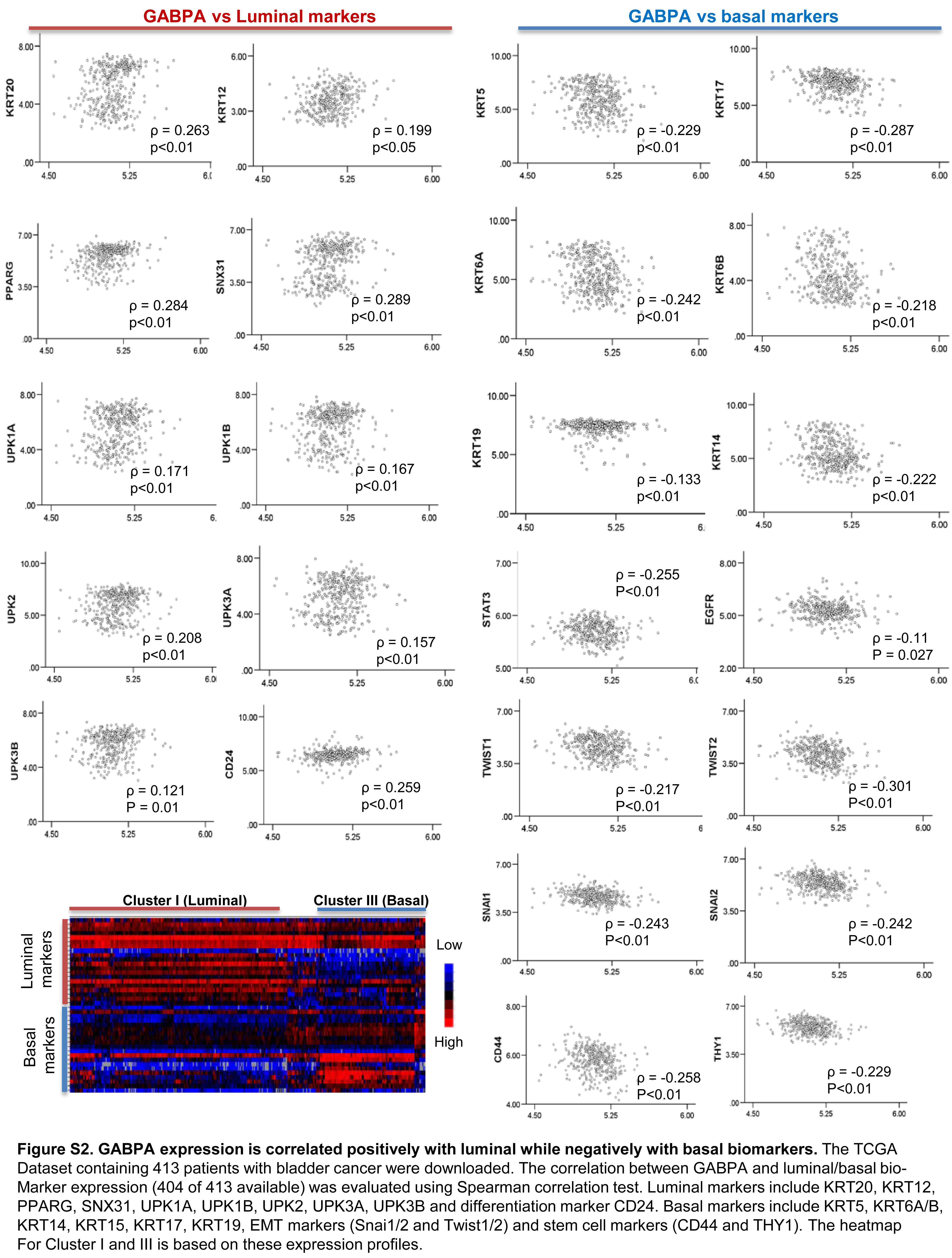

Supplement: Supplementary file 6 — Fig. S2 [file 41418_2019_466_MOESM6_ESM.png]

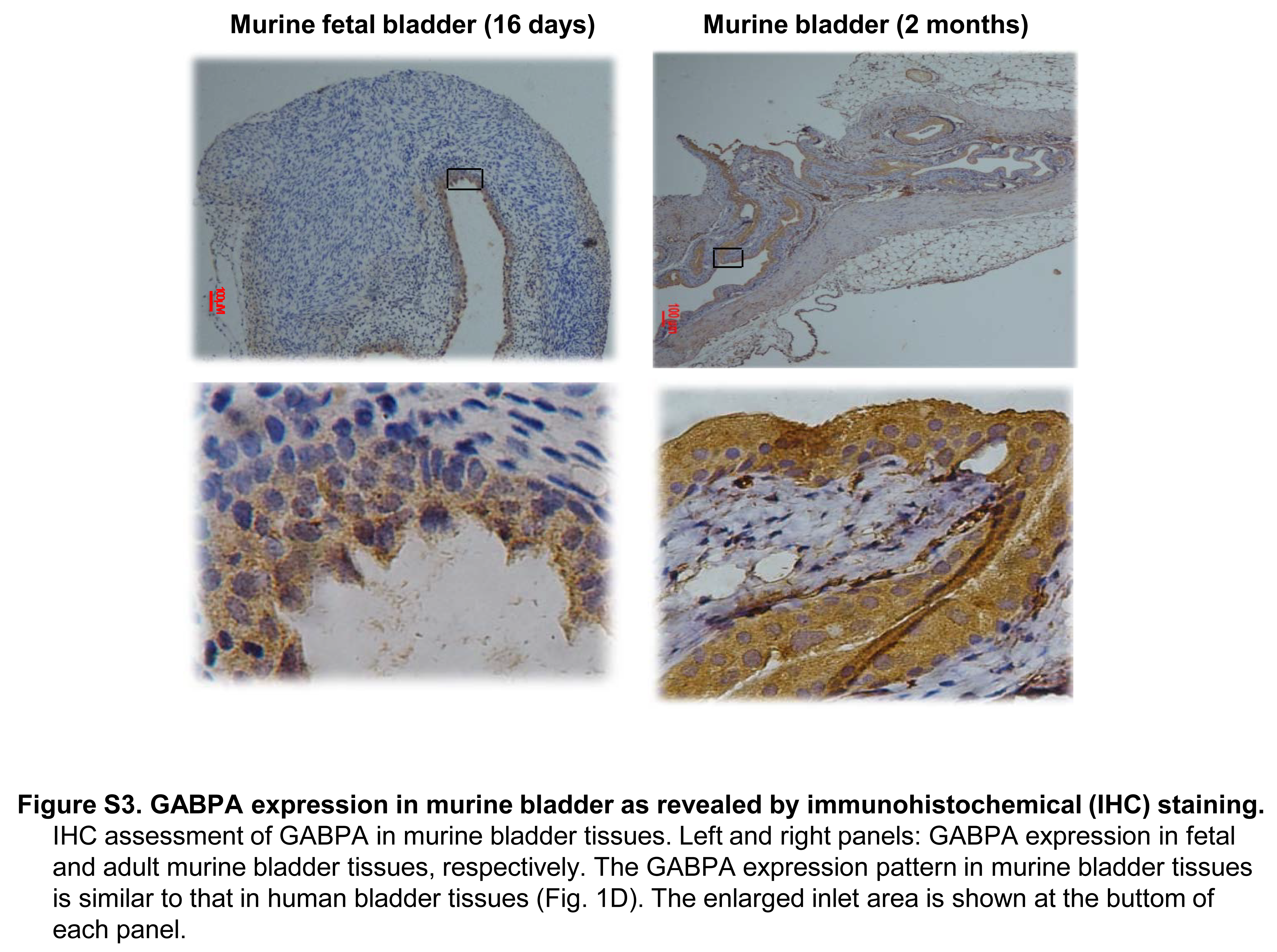

Supplement: Supplementary file 7 — Fig. S3 [file 41418_2019_466_MOESM7_ESM.png]

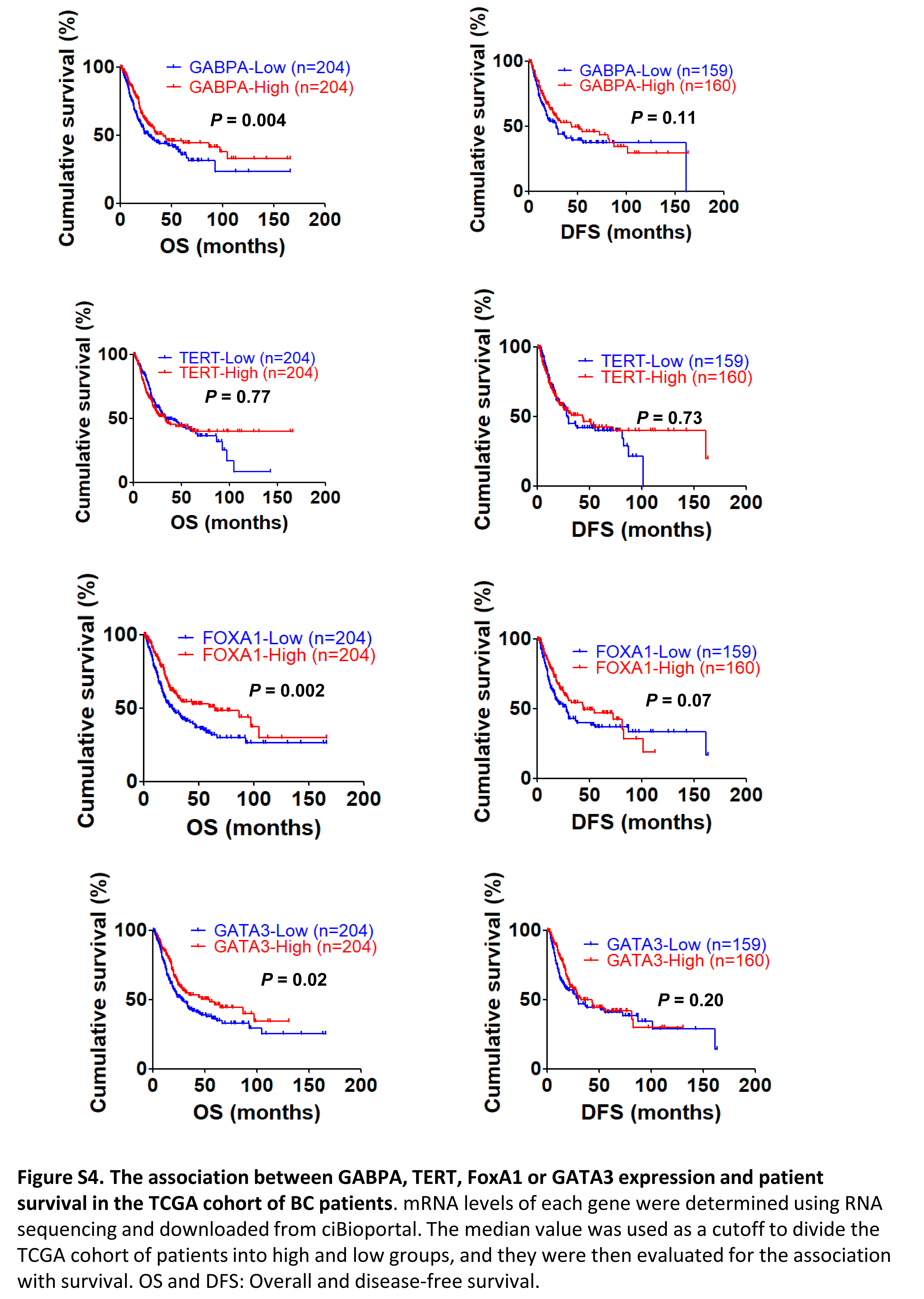

Supplement: Supplementary file 8 — Fig. S4 [file 41418_2019_466_MOESM8_ESM.png]

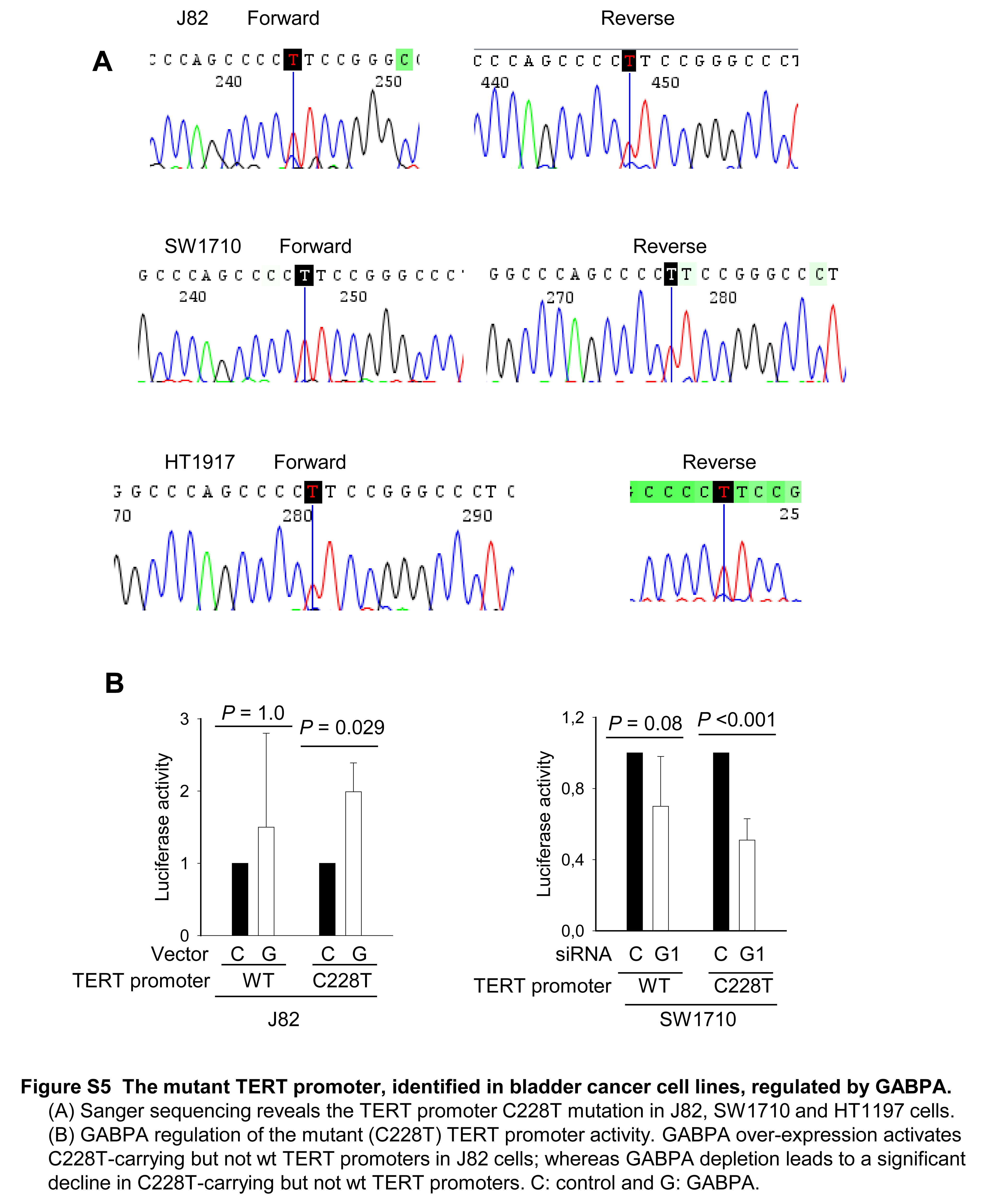

Supplement: Supplementary file 9 — Fig. S5 [file 41418_2019_466_MOESM9_ESM.png]

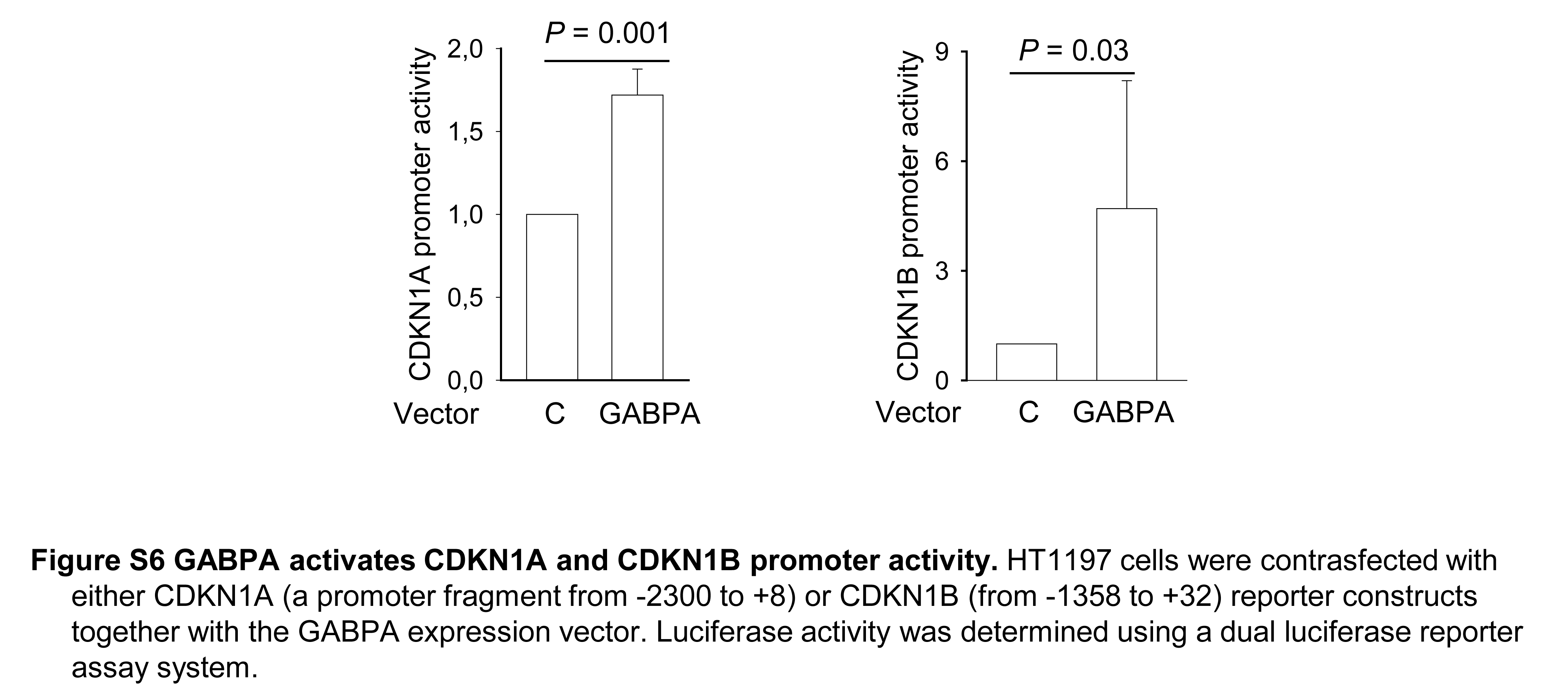

Supplement: Supplementary file 10 — Fig. S6 [file 41418_2019_466_MOESM10_ESM.png]

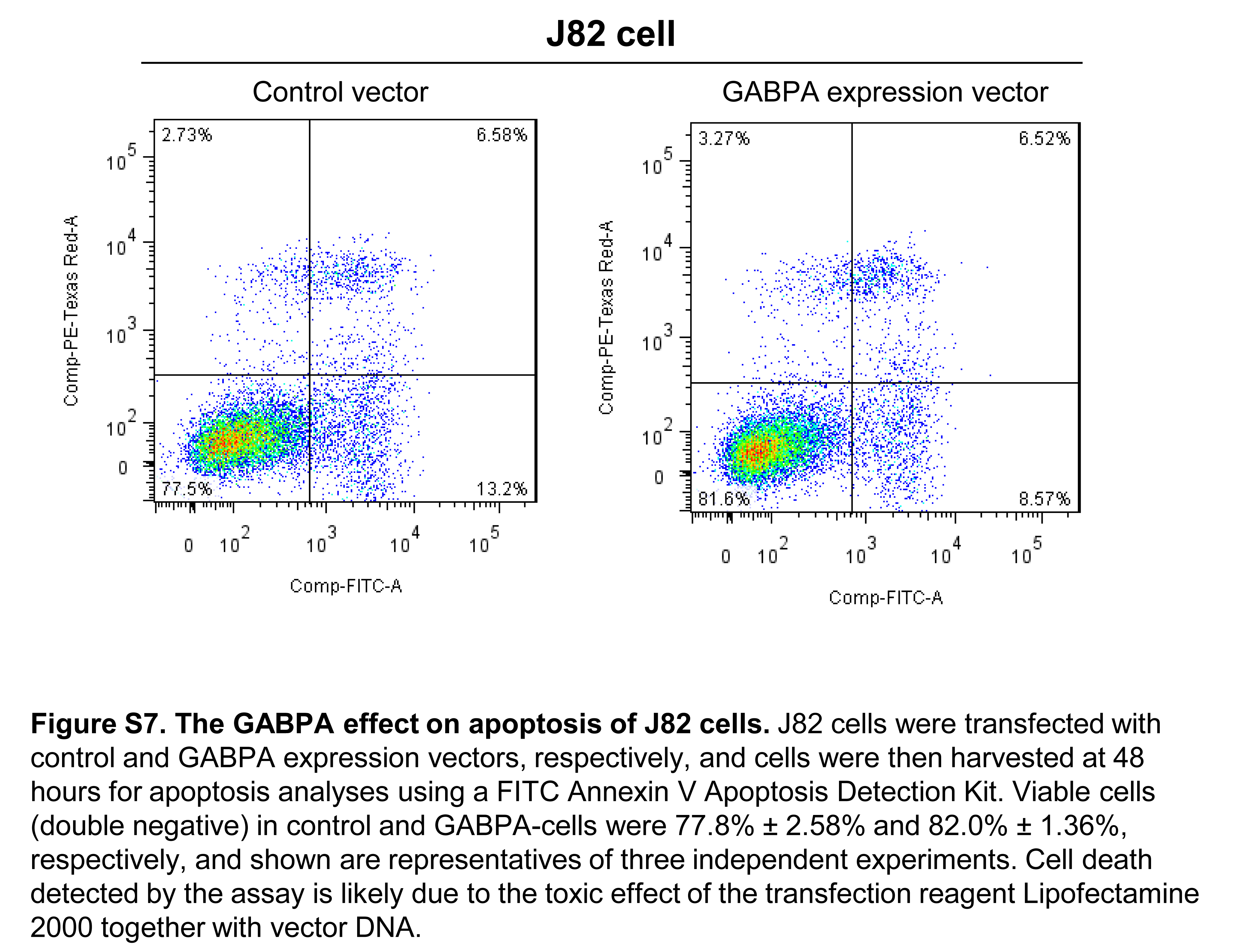

Supplement: Supplementary file 11 — Fig. S7 [file 41418_2019_466_MOESM11_ESM.png]

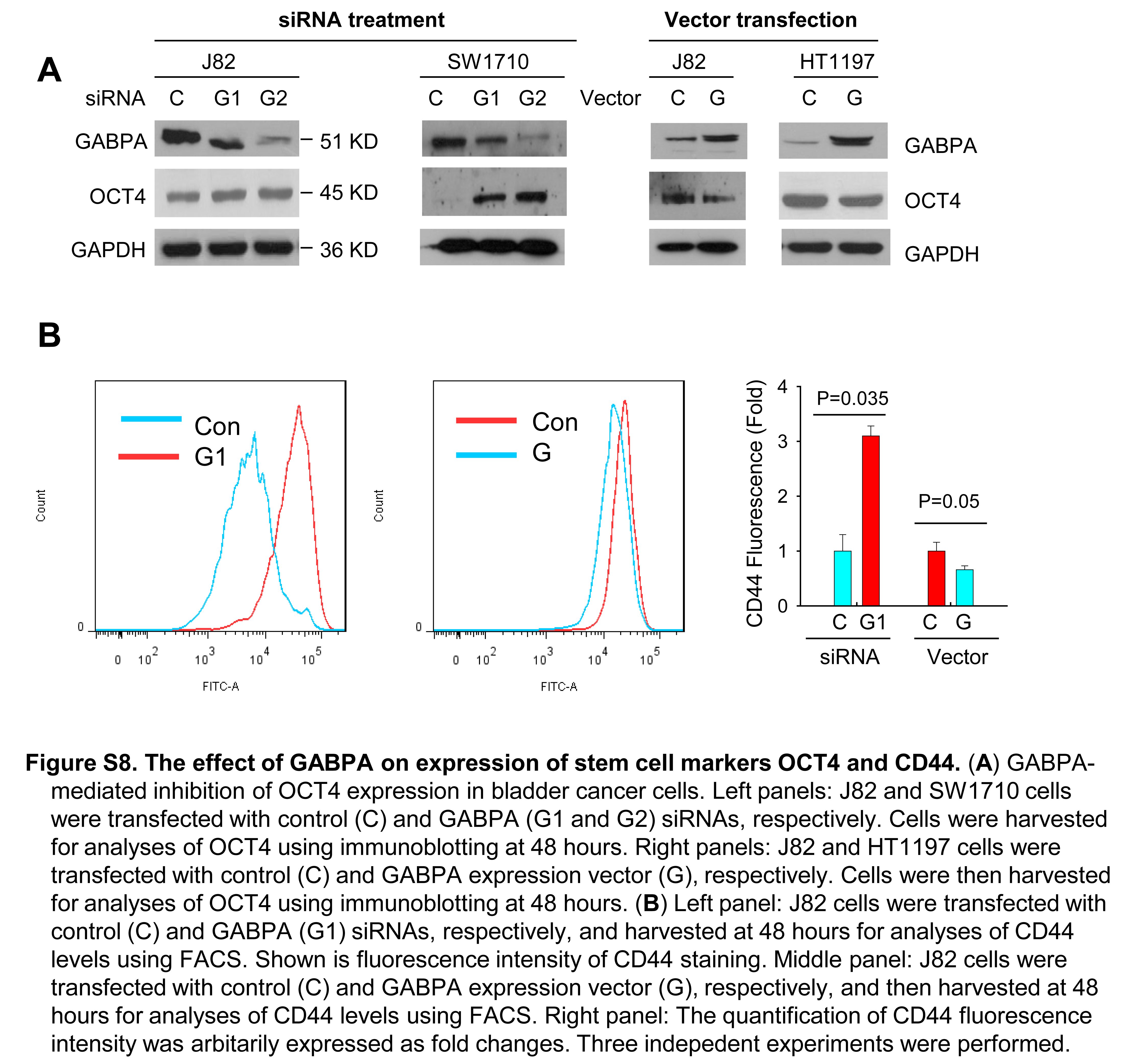

Supplement: Supplementary file 12 — Fig. S8 [file 41418_2019_466_MOESM12_ESM.png]

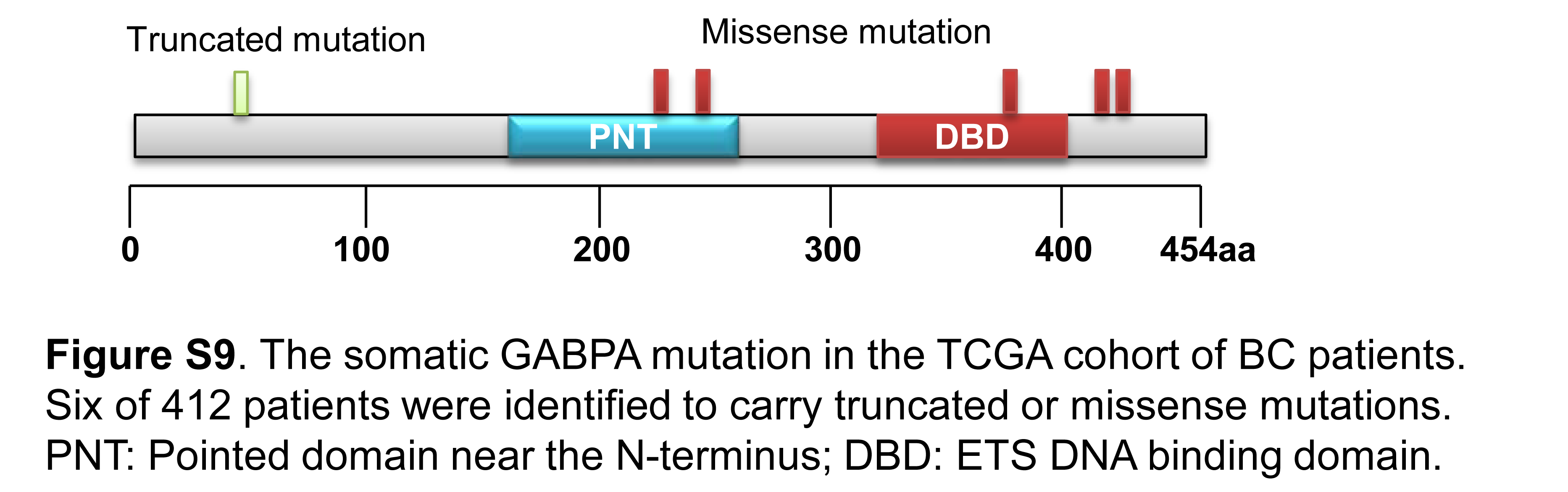

Supplement: Supplementary file 13 — Fig. S9 [file 41418_2019_466_MOESM13_ESM.png]
